# Genetic overlap between birthweight and adult cardiometabolic diseases has implications for genomic medicine

**DOI:** 10.1038/s41598-019-40834-w

**Published:** 2019-03-11

**Authors:** Fasil Tekola-Ayele, Anthony Lee, Tsegaselassie Workalemahu, Wei Zhang, Deepika Shrestha, Azmeraw T. Amare, Marion Ouidir

**Affiliations:** 1 Epidemiology Branch, Division of Intramural Population Health Research, Eunice Kennedy Shriver National Institute of Child Health and Human Development, National Institutes of Health, Bethesda, MD USA; 2 Biostatistics and Bioinformatics Branch, Division of Intramural Population Health Research, Eunice Kennedy Shriver National Institute of Child Health and Human Development, National Institutes of Health, Bethesda, MD USA; 3 School of Medicine, University of Adelaide, Adelaide, SA Australia

## Abstract

Before implementing therapeutic genomic interventions for optimizing health in early life, comprehensive understanding of their effect on several traits across the life course is warranted. Abnorml  birthweight is associated with cardiometabolic disease risk in adulthood; however, the extent of genetic pleiotropy in the association has not been comprehensively investigated. We tested for pleiotropy and enrichment of functional loci between birthweight and 15 cardiometabolic disease traits (CMD). We found significantly abundant genetic pleiotropy (*P* < 3.3 × 10^−3^) and enrichment of functional annotations (*P* < 3.3 × 10^−3^) in loci influencing both birthweight and CMD. We did not observe consistent effect directions of pleiotropic loci on the traits. A total of 67 genetic loci, of which 65 loci have been reported in previous genome-wide association studies, were associated with both birthweight and CMD at a false discovery rate of 5%. Two novel loci were associated with birthweight and adult coronary artery disease (rs2870463 in *CTRB1*) and with birthweight and adult waist circumference (rs12704673 in *CALCR*). Both loci are known to have regulatory effects on expression of nearby genes. In all, our findings revealed pervasive genetic pleiotropy in early growth and adulthood cardiometabolic diseases, implying the need for caution when considering genetic loci as therapeutic targets.

## Introduction

In our rapidly evolving era of genomic medicine, designing genetically-based interventions for a disease in early life, for instance through genome editing approaches^1^, demands a comprehensive understanding of its downstream consequences in later life^2^. In particular, pleiotropic genetic variants^3–5^ that reduce the risk of one disease may be associated with increased or reduced risk of other diseases^6^. Effective and safe implementation of genomic medicine warrants in-depth understanding of the extent of pleiotropy, identification of genetic loci with pleiotropic effects, and their relative directions of effects on diverse human traits across the life course^2^.

Genetic studies linking early life growth traits to later-life cardiometabolic diseases have gained traction because a large number of observational studies have found that birthweight is associated with cardiovascular and metabolic diseases in adulthood^7–13^. Importantly, recent genome-wide association studies (GWAS) have revealed that genetic factors that influence multiple phenotypes contribute to a substantial proportion of the correlations between birthweight and adulthood traits such as waist circumference, body mass index (BMI), type 2 diabetes, and coronary artery disease^14,15^. Specifically, birthweight exhibits significant inverse genetic correlations with type 2 diabetes, fasting insulin, glycated hemoglobin, waist-to-hip ratio, coronary artery disease, high-density lipoprotein cholesterol (HDL), low-density lipoprotein cholesterol (LDL), total cholesterol, and total glycerides, and significant positive genetic correlations with BMI and waist circumference^14,15^. Scientific understanding of genetic pleiotropy among early and later life traits will help unravel common mechanisms that underlie fetal growth aberrations and adult chronic diseases. It may help elucidate molecular functions of genetic variants in different tissues, distinguish biological processes that operate in early life from those that operate in adulthood, and formulate possible causal relationships between traits^16^. Ultimately, knowledge gained from these studies will be critical to rigorously evaluate whether genome-based interventions to optimize fetal and neonatal health are also beneficial in adulthood and do not rather contribute to increased cardiometabolic disease risk.

Although shared genetic loci have been considered to contribute to the life course associations between birthweight and cardiometabolic diseases, to date, the contributions of genetic pleiotropy to the associations of early-life traits with adult disease traits has not been comprehensively studied. Large scale GWASs conducted on several traits measured at birth and during adulthood have provided summary statistics data, paving the way for newly developed statistical approaches to test for genetic pleiotropy^17^.

In the present study, we comprehensively tested for genetic pleiotropy and enrichment of functional genetic loci influencing birthweight and 15 adult cardiometabolic disease traits (CMD) including BMI, waist-to-hip ratio, waist circumference, type 2 diabetes, fasting plasma glucose, fasting plasma insulin, glycated hemoglobin, insulin secretion, insulin sensitivity, coronary artery disease, myocardial infarction, HDL, LDL, total cholesterol, and total glycerides using a statistical approach that integrates pleiotropy and functional annotation data. Our analysis showed evidence for pervasive pleiotropy, and enrichment of functionally annotated loci shared between birthweight and adult cardiometabolic diseases. We also identified genetic variants associated with both birthweight and CMD, of which most are known GWAS signals and two were novel loci (suggestively associated but not at genome-wide significance threshold in previous GWAS) with important regulatory effects on nearby genes.

## Results

### Abundant genetic pleiotropy between birthweight and adult cardiometabolic disease traits

Tests for genetic pleiotropy were performed between birthweight (as a continuous trait accounting for gestational age and offspring sex) and each of the individual CMD using a unified statistical approach implemented in Genetic analysis incorporating Pleiotropy and Annotation (GPA)^18^. Pleiotropic genetic effects were significant between birthweight and each of the 15 CMD after Bonferroni correction (*P* < 3.33 × 10^−3^) (Table 1 and S1 and S2). The three CMD that shared the largest proportion of genetic loci associated with birthweight were BMI (6.21%), type 2 diabetes (4.37%), and total cholesterol (3.60%) (Table S3).

**Table 1:** Genetic pleiotropy and enrichment of functional deleteriousness among genetic loci associated with birthweight and adult cardiometabolic disease traits.

Cardiometabolic disease traits (CMD)	Genetic pleiotropy		Functional annotation enrichment	
	Enrichment fold (s.e.)	*P*-value	*q*_11_/*q*_00_ (s.e.)	*P*-value
Coronary artery disease	8.61 (0.09)	<10^−300^	1.25 (0.06)	6.87 × 10^−22^
Myocardial infarction	8.13 (0.11)	<10^−300^	1.14 (0.08)	1.01 × 10^−18^
Type 2 diabetes	14.14 (0.28)	<10^−300^	1.45 (0.09)	1.73 × 10^−21^
Fasting glucose	8.22 (0.97)	4.58 × 10^−24^	0.47 (0.99)	0.44
Fasting insulin	3.69 (0.16)	3.52 × 10^−77^	1.15 (0.21)	1.46 × 10^−21^
Hemoglobin A1c	5.37 (0.16)	2.35 × 10^−233^	1.29 (0.15)	1.63 × 10^−22^
Insulin secretion	7.39 (0.60)	1.91 × 10^−110^	1.29 (0.29)	1.66 × 10^−21^
Insulin sensitivity	6.89 (0.38)	8.90 × 10^−110^	1.13 (0.26)	9.00 × 10^−23^
HDL cholesterol	6.39 (0.11)	<10^−300^	1.66 (0.09)	1.31 × 10^−28^
LDL cholesterol	4.92 (0.11)	<10^−300^	1.72 (0.11)	5.61 × 10^−33^
Total cholesterol	4.67 (0.09)	<10^−300^	1.66 (0.09)	8.39 × 10^−36^
Triglycerides	4.78 (0.11)	<10^−300^	1.48 (0.13)	2.78 × 10^−27^
Waist circumference	5.66 (0.09)	<10^−300^	1.45 (0.08)	2.7 × 10^−40^
Body mass index	3.94 (0.05)	<10^−300^	1.42 (0.06)	3.72 × 10^−54^
Waist-to-hip ratio	7.05 (0.12)	<10^−300^	1.86 (0.05)	3.66 × 10^−40^

*q*_11_/*q*_00_ is the ratio of the probability of jointly associated SNPs being functionally annotated to the probability of a null SNP (not associated with neither trait) being functionally annotated.

### Enrichment of functional annotations between birthweight and adult cardiometabolic disease traits

**Figure 1:**
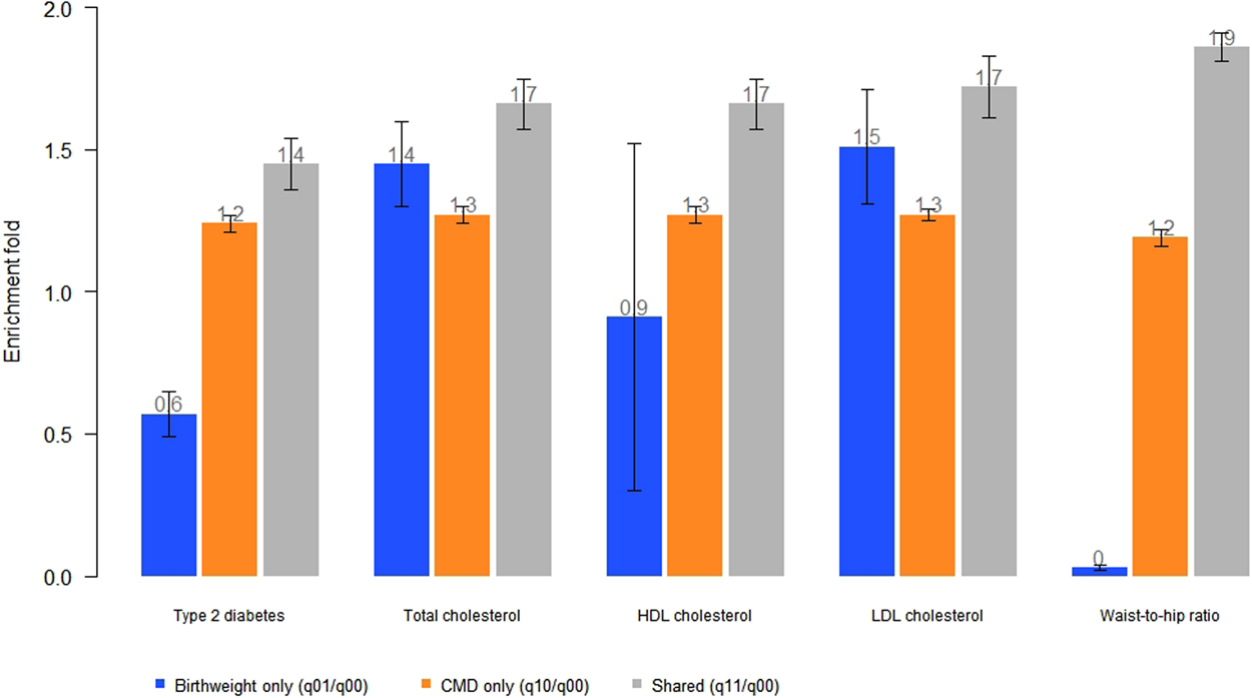
Enrichment of functional annotations for variants associated with birthweight and cardiometabolic disease traits in later life. A vertical line on each bar represents standard error.

In order to test whether genetic loci with known biological function are more likely to be associated with both birthweight and CMD compared to genetic loci that are not functional, tests of functional enrichment were performed between birthweight each of the 15 CMD using GPA. Functional annotation of SNPs was done using the Combined Annotation Dependent Depletion (CADD) score^19^. In 14 out of 15 birthweight-CMD tests (except fasting plasma glucose), single nucleotide polymorphisms (SNPs) associated with both birthweight and CMD were more likely to be functionally deleterious (CADD score >15) than SNPs associated with neither (q_11_/q_00_ ranging from 1.13 to1.86; *P* < 3.33 × 10^−3^) (Tables 1 and S2). Enrichment of functional deleteriousness was stronger for SNPs associated with both birthweight and CMD than SNPs associated only with birthweight or only with CMD in five birthweight-CMD pairs (HDL, LDL, total cholesterol, type 2 diabetes, and waist-to-hip ratio). For these five trait pairs, SNPs associated with birthweight-CMD *vs*. SNPs associated with birthweight only *vs*. SNPs associated with CMD only had the following enrichment folds (*s*.*e*.): 1.66 (0.09) *vs*. 0.91 (0.61) *vs*. 1.27 (0.03) for HDL; 1.72 (0.11) *vs*. 1.51 (0.20) *vs*. 1.27 (0.02) for LDL; 1.66 (0.09) *vs*. 1.45 (0.15) *vs*. 1.27 (0.03) for total cholesterol; 1.45 (0.09) *vs*. 0.57 (0.98) *vs*. 1.24 (0.03) for type 2 diabetes; and 1.86 (0.05) *vs*. 0.03 (12.85) *vs*. 1.20 (0.03) for waist-to-hip ratio (Fig. 1, Table S2).

### Directions of effect of genetic variants on birthweight and adult cardiometabolic disease traits

Variants associated with birthweight and CMD had varying direction of effects on adult traits. Of the genetic loci associated with birthweight and a CMD, all loci associated with lower birthweight were associated with increased waist-to-hip ratio, triglycerides, type 2 diabetes risk, myocardial infarction risk, and coronary artery disease risk. All genetic loci associated with lower birthweight were associated with decreased waist circumference. Conversely, loci associated with lower birthweight showed varied directions of effect on BMI, HDL, LDL, and total cholesterol (Table S4).

### Genetic loci with pleiotropic effect on birthweight and adult cardiometabolic disease traits

**Figure 2:**
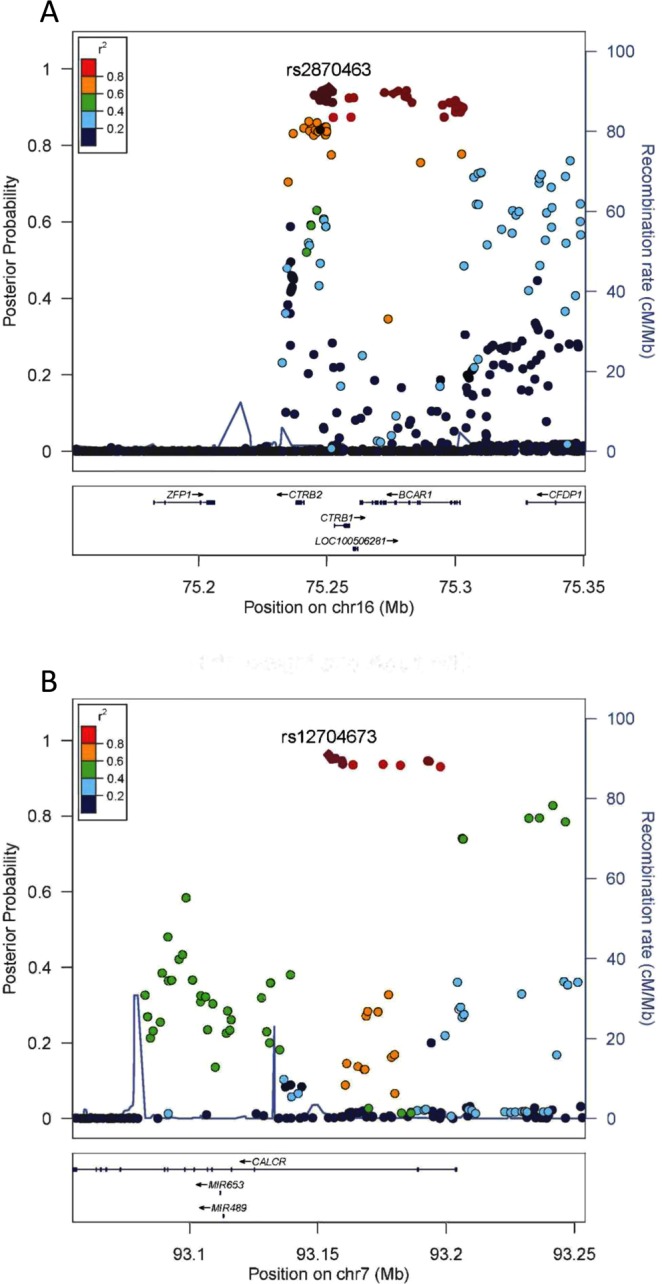
Regional plots of loci associated with birthweight and cardiometabolic disease traits in later life. Data span 200 kb centered at the index SNP. The x-axis denotes genomic position and the y axis denotes the posterior probability of association, and recombination rate (cM/Mb). The purple circle point represents the index SNP. The color of each data point indicates its linkage disequilibrium value (r^2^) with the index SNP based on HapMap2. (**A**) Regional association plot for a locus in *CTRB1* associated with birthweight and coronary artery disease. **(B**) Regional association plot for a locus in *CALCR* associated with birthweight and waist circumference.

A total of 67 loci were associated with birthweight and at least one of 13 CMD (Table S5). Of the 67 loci, 65 map to previously known GWAS signals associated with birthweight or CMD (*P* < 5 × 10^−8^ in the NHGRI-EBI GWAS catalogue: www.ebi.ac.uk/gwas/). Two of the 67 loci (rs2870463 and rs12704673) were only suggestively associated (5 × 10^−8^ < *P* < 5 × 10^−5^) with birthweight, waist circumference, and coronary artery disease in previous GWAS^20,21^. In our study, rs2870463 G in *CTRB1* gene was associated with decreased birthweight and increased risk of coronary artery disease with posterior probability (*PP*) = 0.951 and rs12704673 T in *CALCR* gene was associated with increased birthweight and increased waist circumference with *PP* = 0.962 (Fig. 2). In further functional follow-up analysis, we observed that rs2870463 was *cis*-eQTL with genes in the *BCAR1-CFDP1-TMEM170A* locus in adipose tissue, heart, and whole blood, and overlaps with enhancer histone marks and DNase hypersensitive sites in placenta and pancreas (Table S6). In addition, rs12704673 was also implicated in motif changes of the TCF4 transcription factor (Table S6).

## Discussion

A new era of genomic medicine has been ushered by advances in genome sequencing technologies and dramatic improvements in the efficiency of molecular gene editing tools^1^. With increasing evidence that some genetic variants can have pleiotropic effects on two or more phenotypes^3–5^, it is possible that therapeutic genetic interventions for treating one disease may have beneficial or untoward consequences on other phenotypes across the life span. The present study investigated genetic pleiotropic effects between birthweight and a scope of adult metabolic and cardiovascular traits and revealed that shared genetic influence is a common phenomenon. We also found that biologically functional SNPs are more likely to be associated with both birthweight and later life traits compared to SNPs that are not predicted to be functional, consistent with previous observation of high evolutionary conservation of pleiotropic genes and their consequences^22^. Lastly, we identified pleiotropic effects at 65 genetic loci associated with either birthweight or CMD in previous GWAS and at two loci (in *CALCR* and near *CTRB1*) that were only suggestively associated with both traits in previous GWAS. The study facilitates our understanding of the genetic mechanisms that underlie associations of early life growth with later life cardiometabolic traits, reproducibly shown in several epidemiological studies^7–13^.

We observed that pleiotropic variants associated with lower birthweight may increase or decrease risk for adult cardiometabolic diseases. Variants that reduced birthweight increased the risk of myocardial infarction and coronary artery disease, suggesting that convergent genetic mechanisms may play important roles in the well-known inverse correlation between low birthweight and adult cardiovascular diseases^23^. Our study also found that birthweight-decreasing alleles may be associated with either increased to decreased adult lipid and adiposity traits. Previous studies have reported that type 2 diabetes risk alleles or blood pressure raising alleles may be associated with either higher or lower birthweight^15,24^. Genetic loci near *NT5C2*, *FES*, *NRIP1*, *EBF1* and *PTH1R* genes have been associated with higher systolic blood pressure and lower birthweight, whereas a locus near *ADRB1* has been found to be associated with higher systolic blood pressure and birthweight. The variant near *NT5C2* has also been found to be a proxy for a functional variant in *CYP17A1* gene that is expressed in fetal tissues including the placenta, with potential roles in glucocorticoid synthesis^15^. Similarly, while the type 2 diabetes risk loci in *ADCY5*, *CDKAL1* and *HHEX-IDE* were associated with lower birthweight, type 2 diabetes risk loci in *ANK1* and *MTNR1B* were associated with higher birthweight^15^. For type 2 diabetes, these findings support observational studies that found that individuals at either extreme of the birthweight distribution have greater risk for type 2 diabetes in adulthood^25^.

Therefore, mixed directions of effect of the pleiotropic loci found in our study highlight the complexity in early-later life trait relationships, and may provide new clues to understand the mechanisms of development of adult diseases. The findings further caution that a targeted functional study of these loci is warranted to resolve their downstream phenotypic consequences if the loci are targeted by genomic medicine, for example through gene editing or pharmacogenomics. Moreover, we observed that several loci had potential *cis*-regulatory effect on genes that operate in a wide range of tissues. This finding further supports the possibility that the associated loci may have multiple downstream consequences. This observation is also consistent with a previous study in yeast that found that pleiotropic effects of a gene are usually conferred by multiple consequences of a single molecular function^22^.

The differences in the relative fraction of genetic loci shared by birthweight and later life traits suggested that the contribution of genetics to the life course association between early and later life traits is complex. Only a small fraction (<5%) of birthweight loci were associated with adult traits whereas most of the adult trait loci were associated with birthweight (Table S3). These findings highlight the complexity of the genetic architecture of fetal growth, in which, a complex array of genetic variants that play roles in many adulthood diseases as well as some that uniquely influence life *in utero* are implicated in fetal growth. This is consistent with and extends previous observations of shared genetic background between birthweight and type 2 diabetes and hypertension^15,24^.

It is noteworthy that rs2870463 G near *CTRB1* and *BCAR1* was found to be significantly associated with decreased birthweight and higher risk of coronary artery disease. No previous genome-wide significant associations of the locus with coronary artery disease have been identified; however, *BCAR1-CFDP1-TMEM170A* locus (just 80 kb from our novel SNP) has been found to be suggestively associated with and functionally implicated in carotid intima-media thickness^26,27^, and had genome-wide significant association with aortic root diameter (a marker of cardiac structure)^28^. This novel pleiotropic SNP (rs2870463) found in the present study is in strong LD (r^2^ = 1) with an intronic SNP in *BCAR1* (rs7202877), which has previously been implicated in impaired B-cell function^29^. In addition, rs2870463 had *cis*-regulatory effect on expression of *BCAR1* and *CFDP1* in blood and overlaps with promotor histone marks and DNAse hypersensitive sites in the pancreas and placenta. Several studies have demonstrated that impairment of pancreatic B-cell function is associated with development of coronary artery disease independent of glucose metabolism^30,31^ and with reduced birthweight^32–34^. Together, these findings indicate a potential functional role of the associated SNP in regulating pancreatic B-cell function by mediating expression of *BCAR1* and the locus may underlie previously observed inverse associations between birthweight and cardiovascular outcomes^23^.

Moreover, rs12704673 in *CALCR* associated with birthweight and adult waist circumference has not been associated with waist circumference in previous GWAS, but has shown genome-wide significant associations with other adiposity/anthropometric traits such as BMI and hip circumference in adults^20,35,36^. *CALCR* encodes the calcitonin receptor protein that is involved in maintaining calcium homeostasis. Our study also found that *CALCR* as well as two other genes previously known to be associated with BMI in adults (*SCARB2* and *KLF3-AS1*)^35,37^ had joint influence on birthweight and adult BMI. These three loci are notable because to date, no locus has been reported to be associated with both birthweight and adiposity traits at a genome-wide significance. Functional studies of these loci may unlock biological pathways of adiposity that begin early in life.

We acknowledge that our study has limitations. Despite the large sample sizes of the consortia-based meta-analysis studies included in our study, there were differences in sample size and number of SNPs among the different studies. These differences may contribute to study power differences in identifying pleiotropic loci. In addition, some of the observed associations may not be due to independent effects of the same locus on birthweight and a later life trait, but because of correlation of the traits in a causal pathway or through other unmeasured traits. Our analysis did not identify significant pleiotropy between birthweight and fasting glucose, which requires further investigation in larger samples, but is consistent with a previous study that did not find significant genetic correlation between birthweight and fasting glucose^15^. An important strength of our study is the integrated modelling of functional annotation and GWAS summary statistics data from pairs of traits. This multi-trait approach has been instrumental in testing for functional enrichment and detection of novel loci with multi-trait effects, considerably expanding our limited understanding of the genetic links between fetal growth and later life traits.

In conclusion, this study found pervasive pleiotropic genetic effects and significant enrichment of functional annotations in genetic variants jointly associated with birthweight and later life cardiometabolic traits and diseases. The novel loci identified in the study and the pathways through which the genes operate bear potential to disentangle the genetic basis of life course associations between early growth and adulthood cardiometabolic diseases. The complex directions of effect of pleiotropic loci indicates that responsible cautions should be taken in genomic medicine, to minimize untoward later-life consequences of therapeutic genomics such as gene editing in early life.

## Methods

### Data sets

GWAS summary statistics data including *P*-values and directions of effect of genome-wide SNPs for birthweight and CMDs including BMI, waist-to-hip ratio, waist circumference, type 2 diabetes, fasting plasma glucose, fasting plasma insulin, glycated hemoglobin, insulin secretion, insulin sensitivity, coronary artery disease, myocardial infarction, LDL, HDL, total cholesterol, and total glycerides were assembled from reports of six consortia^15,20,21,37–43^ (Table S1). The Consortia included the Early Growth Genetics Consortium (EGG, http://egg-consortium.org); Genetic Investigation of Anthropometric Traits (GIANT, http://portals.broadinstitute.org/collaboration/giant/index.php/GIANT_consortium); DIAbetes Genetics Replication And Meta-analysis (DIAGRAM, http://www.diagram-consortium.org); Meta-Analysis of Glucose and Insulin-related traits Consortium (MAGIC, https://www.magicinvestigators.org/); Coronary Artery Disease Genomewide Replication and Meta-analysis (CARDIoGRAM) plus The Coronary Artery Disease (C4D) Genetics Consortium (http://www.cardiogramplusc4d.org/); and Global Lipids Genetics Consortium (GLGC, http://lipidgenetics.org). The majority of the study participants were individuals of European ancestry and the sample sizes ranged from 5,318 to 339,224^15,20,21,37–43^. More details about the datasets are presented in Table S1.

Informed consent was obtained from participating individuals and the respective institutional ethics review boards approved the studies. The NIH Office of Human Subjects Research Program granted the study an exemption from review by an institutional review board per 45 CFR 46 on the use of specimens and data.

We tested for evidence of pleiotropy, enrichment of functional annotation, and association of SNPs with both birthweight and CMD using the GPA v1.1-0 R package^18^. GPA implements a unified statistical approach that integrates pleiotropy and functional annotation data, and tests for enrichment of annotations in variants associated with pairs of traits. A total of 15 birthweight-CMD pair tests were performed.

### Tests for genetic pleiotropy and enrichment of functional annotation

All tests were conducted under the false discovery rate control (FDR) at the 0.05 level using 10,000 Expectation-Maximization (EM) iterations. Evidence for enrichment of pleiotropy and functional annotation were considered significant at the Bonferroni-corrected level *P*-value = 3.33 × 10^−3^ (0.05/15 tests). An FDR cutoff of 0.05 was used to identify SNPs that were significantly associated with both traits in a birthweight-CMD pair^18^. When two or more SNPs within a 1 Mb region were associated with a trait-pair, the index SNP with the highest posterior probability of association and other SNPs not in linkage disequilibrium (LD) with the index SNP (r^2^ < 0.06 in the 1000 Genomes Phase 3 Utah Residents with Northern and Western European ancestry (CEU) population sample), were considered to be independent associations.

Functional annotation of SNPs was carried out using the Combined Annotation Dependent Depletion (CADD) framework as implemented in CADD v1.2 (http://cadd.gs.washington.edu)^19^. CADD integrates functional and evolutionary importance from multiple annotation sources to generate a deleteriousness score for each genetic variant. Variants with Phred-like CADD score (−10*log10 [rank/total]) values ≥ 15 were considered deleterious^19^, and were assigned annotation of 1 and those with CADD score values < 15 were assigned annotation of 0. The assigned annotation values were used as inputs for GPA. Subsequent annotation tests assessed functional enrichment among SNPs associated only with birthweight, compared to SNPs associated with neither trait (estimated by q_10_/q_00_), among SNPs associated only with CMD, compared to SNPs associated with neither trait (q_01_/q_00_), and among SNPs associated with both birthweight and CMD, compared to SNPs associated with neither trait (q_11_/q_00_). As implemented in GPA^18^, an efficient EM algorithm estimated model parameters and the standard errors of the parameters were calculated from covariance matrix derived from the empirical observed information matrix. The likelihood ratio test was used to assess the significance of enrichment of the annotations. SNPs were sorted based on their local FDR from the smallest to the largest, and the direct posterior probability approach was used to control global FDR to determine associated SNPs^18^.

### Functional follow-up of lead SNPs

Further functional genomic analyses of SNPs newly identified to be associated with birthweight and CMD was performed using the Genotype-Tissue Expression (GTEx v. 6)^44^ database for potential regulatory effect on gene expression level in different tissues. Possible regulatory effects of the lead SNPs were assessed by examining if the SNPs are within promotors, enhancers, DNAse, and transcription factor binding using the Haploreg tool (version 4.1)^45^.

## Supplementary information

Supplementary Information

## Data Availability

The data analyzed in this study are available online. Table S1 lists the URL of the data sources.
