# Supplementary material for: Genetic overlap between birthweight and adult cardiometabolic diseases has implications for genomic medicine
Source: Sci Rep. 2019 Mar 11;9:4076. doi: 10.1038/s41598-019-40834-w (PMC6411883; doi:10.1038/s41598-019-40834-w)
Supplement: Supplementary file 1 — Supplementary Information [file 41598_2019_40834_MOESM1_ESM.docx]

**Supplementary Information**

**Genetic overlap between birthweight and adult cardiometabolic diseases has implications for genomic medicine**

Fasil Tekola-Ayele^1*^, Anthony Lee^1^, Tsegaselassie Workalemahu^1^, Wei Zhang^2^, Deepika Shrestha^1^, Azmeraw T. Amare^3^, Marion Ouidir^1^

^1^ Epidemiology Branch, Division of Intramural Population Health Research, *Eunice Kennedy Shriver* National Institute of Child Health and Human Development, National Institutes of Health, Bethesda, MD, USA

^2^ Biostatistics and Bioinformatics Branch, Division of Intramural Population Health Research, *Eunice Kennedy Shriver* National Institute of Child Health and Human Development, National Institutes of Health, Bethesda, MD, USA

^3^ School of Medicine, University of Adelaide, Adelaide, SA, Australia

*Correspondence:*

*Fasil Tekola-Ayele, PhD

Epidemiology Branch, Division of Intramural Population Health Research,

*Eunice Kennedy Shriver* National Institute of Child Health and Human Development

National Institutes of Health

6710B Rockledge Drive, Room 3204

Bethesda, MD 20892-7004

E-mail: ayeleft@mail.nih.gov

Tel: 301-827-6518

**Table S1.** GWAS summary statistics profile of birth weight and adult cardiometabolic disease traits analyzed in the study.

| **Trait** | **Source/Consortium** | **Sample size** | **Number of SNPs** | **PubMed ID (Reference)** |
| --- | --- | --- | --- | --- |
| Birth weight | Early Growth Genetics Consortium (EGG);  <http://egg-consortium.org> | 153,781 | 22,411,934 | 27680694 ((1)) |
| Body mass index | Genetic Investigation of Anthropometric Traits (GIANT); <http://portals.broadinstitute.org/collaboration/giant/index.php/GIANT_consortium> | 339,224 | 2555510 | 25673413 ((2)) |
| Waist-hip ratio |  | 224,459 | 2598692 | 25673412 ((3)) |
| Waist circumference |  | 224,459 | 2601994 | 25673412 ((3)) |
| Type 2 diabetes | DIAbetes Genetics Replication And Meta-analysis (DIAGRAM);  <http://www.diagram-consortium.org> | 110,452 | 2914607 | 24509480 ((4)) |
| Fasting glucose | Meta-Analysis of Glucose and Insulin-related traits Consortium (MAGIC);  <https://www.magicinvestigators.org/> | 46,186 | 2523878 | 20081858 ((5)) |
| Fasting insulin |  | 46,186 | 2523878 | 20081858 ((5)) |
| Hemoglobin A1C |  | 46,368 | 2598230 | 20858683 ((6)) |
| Insulin secretion- CIR |  | 5,318 | 2488776 | 24699409 ((7)) |
| Insulin sensitivity - ISI |  | 16,753 | 2485212 | 27416945 ((8)) |
| Coronary artery disease | Coronary Artery Disease Genomewide Replication and Meta-analysis (CARDIoGRAM) plus The Coronary Artery Disease (C4D) Genetics Consortium;  <http://www.cardiogramplusc4d.org/> | 184,305 | 9455778 | 26343387 ((9)) |
| Myocardial infarction |  | ~129,000 | 11169182 | 26343387 ((9)) |
| LDL cholesterol | Global Lipids Genetics Consortium;  <http://lipidgenetics.org> | 188,577 | 2437751 | 24097068 ((10)) |
| HDL cholesterol |  | 188,577 | 2447441 | 24097068 ((10)) |
| Total cholesterol |  | 188,577 | 2446981 | 24097068 ((10)) |
| Triglycerides |  | 188,577 | 2439432 | 24097068 ((10)) |

**Table S2.** Genetic pleiotropic effects and enrichment of functional deleteriousness among SNPs associated with both birthweight and cardiometabolic disease traits (CMD) in adulthood

| Cardiometabolic disease or trait | Number of SNPs | pi00 (se) | pi10 (se) | pi01 (se) | pi11 (se) | Enrichment fold (s.e.) | P-value | Functional enrichment among SNPs associated with birthweight compared to SNPs associated with neither trait [q_10_/q_00_ (se)] | Functional enrichment among SNPs associated with CMD compared to SNPs associated with neither trait [q_01_/q_00_ (se)] | Functional enrichment among SNPs associated with both birthweight and CMD compared to SNPs associated with neither trait [q_11_/q_00_ (se)] | Annotation test statistics | P-value |
| --- | --- | --- | --- | --- | --- | --- | --- | --- | --- | --- | --- | --- |
| Body mass index | 2590752 | 0.84 (0.001) | 0.03 (0.001) | 0.10 (0.001) | 0.03 (0) | 3.94 (0.05) | < 10^-300^ | 1.52 (0.07) | 1.31 (0.04) | 1.42 (0.06) | 251.13 | 3.72E-54 |
| Coronary artery disease | 8544005 | 0.88 (0.001) | 0 (0) | 0.10 (0.001) | 0.01 (0) | 8.61 (0.09) | < 10^-300^ | 40.72 (20.19) | 1.18 (0.02) | 1.25 (0.06) | 101.65 | 6.87E-22 |
| Fasting glucose | 2495051 | 0.88 (0.006) | 0 (0.003) | 0.09 (0.006) | 0.03 (0.004) | 8.22 (0.97) | 4.58E-24 | 0.04 (10825.15) | 1.47 (0.24) | 0.47 (0.99) | 2.69 | 0.4407562 |
| Fasting insulin | 2495051 | 0.83 (0.003) | 0.04 (0.003) | 0.09 (0.003) | 0.04 (0.003) | 3.69 (0.16) | 3.52E-77 | 1.37 (0.25) | 1.42 (0.09) | 1.15 (0.21) | 100.12 | 1.46E-21 |
| Hemoglobin A1c | 2566561 | 0.86 (0.001) | 0.01 (0.001) | 0.11 (0.001) | 0.02 (0.001) | 5.37 (0.16) | 2.35E-233 | 1.55 (0.35) | 1.32 (0.04) | 1.29 (0.15) | 104.55 | 1.63E-22 |
| HDL cholesterol | 2481375 | 0.87 (0.001) | 0.001 (0) | 0.12 (0.001) | 0.007 (0) | 6.39 (0.11) | < 10^-300^ | 0.91 (0.61) | 1.27 (0.03) | 1.66 (0.09) | 132.85 | 1.31E-28 |
| Insulin secretion | 2460930 | 0.87 (0.001) | 0 (0) | 0.13 (0.001) | 0.004 (0) | 7.39 (0.60) | 1.91E-110 | 6.21 (1.23) | 1.32 (0.03) | 1.29 (0.29) | 99.87 | 1.66E-21 |
| Insulin sensitivity | 2459303 | 0.86 (0.001) | 0.001 (0.001) | 0.13 (0.001) | 0.01 (0.001) | 6.89 (0.38) | 8.90E-110 | 11.03 (1.07) | 1.34 (0.04) | 1.13 (0.26) | 105.75 | 9E-23 |
| LDL cholesterol | 2471777 | 0.87 (0.001) | 0.003 (0) | 0.13 (0.001) | 0.005 (0) | 4.92 (0.11) | < 10^-300^ | 1.51 (0.20) | 1.27 (0.02) | 1.72 (0.11) | 153.11 | 5.61E-33 |
| Myocardial infarction | 8541959 | 0.88 (0.001) | 0 (0) | 0.11 (0.001) | 0.01 (0) | 8.13 (0.11) | < 10^-300^ | 6.44 (1.11) | 1.19 (0.02) | 1.14 (0.08) | 86.90 | 1.01E-18 |
| Type 2 diabetes | 2893797 | 0.88 (0.001) | 0 (0) | 0.11 (0.001) | 0.01 (0) | 14.14 (0.28) | < 10^-300^ | 0.57 (0.08) | 1.24 (0.03) | 1.45 (0.09) | 99.79 | 1.73E-21 |
| Total cholesterol | 2480907 | 0.88 (0.001) | 0.004 (0) | 0.12 (0.001) | 0.01 (0) | 4.67 (0.09) | < 10^-300^ | 1.45 (0.15) | 1.27 (0.03) | 1.66 (0.09) | 166.21 | 8.39E-36 |
| Triglycerides | 2473236 | 0.87 (0.001) | 0.003 (0) | 0.13 (0.001) | 0.004 (0) | 4.78 (0.11) | < 10^-300^ | 1.61 (0.18) | 1.28 (0.03) | 1.48 (0.13) | 126.70 | 2.78E-27 |
| Waist circumference | 2569939 | 0.86 (0.001) | 0.01 (0) | 0.11 (0.001) | 0.02 (0) | 5.66 (0.09) | < 10^-300^ | 1.98 (0.17) | 1.29 (0.03) | 1.45 (0.08) | 187.01 | 2.7E-40 |
| Waist-to-hip ratio | 2566817 | 0.87 (0.001) | 0.001 (0) | 0.11 (0.001) | 0.02 (0) | 7.05 (0.12) | < 10^-300^ | 0.03 (0.01) | 1.19 (0.03) | 1.86 (0.05) | 186.39 | 3.66E-40 |

**Table S3.** SNPs associated with cardiometabolic disease traits (CMD) in adulthood among those associated with birthweight (at posterior probability >0.95)

| **Adult CMD** | **Number of SNPs tested** | **Proportion of SNPs associated with birthweight that are shared with CMD** |
| --- | --- | --- |
| Coronary artery disease | 8544005 | 0.021 |
| Fasting glucose | 2495051 | 0 |
| Hemoglobin A1c | 2566561 | 0.005 |
| Insulin secretion | 2460930 | 0.013 |
| Insulin sensitivity | 2459303 | 0.004 |
| LDL cholesterol | 2471777 | 0.031 |
| Myocardial infarction | 8541959 | 0.019 |
| Total cholesterol | 2480907 | 0.036 |
| Type 2 diabetes | 2893797 | 0.044 |
| Triglycerides | 2473236 | 0.028 |
| Waist-to-hip ratio | 2566817 | 0.008 |
| Body mass index | 2590752 | 0.062 |
| HDL cholesterol | 2481375 | 0.023 |
| Waist circumference | 2569939 | 0.008 |
| Fasting insulin | 2495051 | NA |

**Table S4.** Direction of effect of genetic variants significantly associated with birthweight and cardiometabolic disease traits in later life (at posterior probability of association >0.95).

| **Adult CMD** | **Total Number of SNPs associated with birthweight and CMD** | **Number (%) associated with decreased birthweight and increased CMD risk/measure** | **Number (%) associated with decreased birthweight and decreased CMD risk/measure** |
| --- | --- | --- | --- |
| Coronary artery disease | 5 | 5 (100%) | 0 |
| Fasting glucose | 0 | 0 | 0 |
| Hemoglobin A1c | 2 | 2 (100%) | 0 |
| Insulin secretion | 3 | 2 (67%) | 1 (33%) |
| Insulin sensitivity | 1 | 0 | 1 (100%) |
| LDL cholesterol | 8 | 4 (50%) | 4 (50%) |
| Myocardial infarction | 4 | 4 (100%) | 0 |
| Total cholesterol | 10 | 5 (50%) | 5 (50%) |
| Type 2 diabetes | 8 | 8 (100%) | 0 |
| Triglycerides | 11 | 11 (100%) | 0 |
| Waist-to-hip ratio | 5 | 5 (100%) | 0 |
| Body mass index | 11 | 4 (33%) | 7 (67%) |
| HDL cholesterol | 16 | 5 (33%) | 11 (67%) |
| Waist circumference | 8 | 0 | 8 (100%) |
| Fasting insulin | 0 | 0 | 0 |

**Table S5.** Genetic loci significantly associated with both birthweight and cardiometabolic disease traits in adulthood (CMD) at posterior probability of association > 0.95

| **CMD** | **SNP** | **Chr** | **Position (hg19)** | **Gene** | **P-value,**  **CMD** | **P-value, Birthweight** | **PP_00_ (null)** | **PP_10_**  **(CMD)** | **PP_01_ (Birthweight)** | **PP_11_**  **(Birthweight-CMD)** |
| --- | --- | --- | --- | --- | --- | --- | --- | --- | --- | --- |
| Body mass index | rs11577179 | 1 | 155983710 | *SSR2* | 3.79E-05 | 3.5E-8 | 2.8198E-05 | 0.00083724 | 0.0325547 | 0.96657986 |
| Body mass index | rs1515098 | 2 | 227073854 | *NYAP2-IRS1* | 8.80E-07 | 2.3E-6 | 2.0311E-05 | 0.00927466 | 0.00216485 | 0.98854018 |
| Body mass index | rs11708067 | 3 | 123065778 | *ADCY5* | 1.99E-05 | 2.8E-26 | 8.9493E-16 | 4.2362E-14 | 0.02068874 | 0.97931126 |
| Body mass index | rs4833079 | 4 | 38654681 | *KLF3-AS1* | 9.40E-06 | 2.7E-6 | 0.00012294 | 0.01005131 | 0.01196016 | 0.97786559 |
| Body mass index | rs17001654 | 4 | 77129568 | *SCARB2* | 5.03E-09 | 0.000033 | 2.1096E-06 | 0.04098266 | 4.9363E-05 | 0.95896587 |
| Body mass index | rs6569648 | 6 | 130349119 | *L3MBTL3* | 1.84E-06 | 3.8E-7 | 1.2519E-05 | 0.00334275 | 0.00371863 | 0.9929261 |
| Body mass index | rs10488550 | 7 | 93155308 | *CALCR* | 8.70E-07 | 4.2E-6 | 2.827E-05 | 0.01301751 | 0.0021387 | 0.98481552 |
| Body mass index | rs12220375 | 10 | 104901491 | *AS3MT-CNNM2* | 1.76E-09 | 7.6E-9 | 8.7072E-09 | 0.00036299 | 2.3978E-05 | 0.99961303 |
| Body mass index | rs3184504 | 12 | 111884608 | *ATXN2* | 4.39E-06 | 3.7E-6 | 8.4954E-05 | 0.01206373 | 0.00690787 | 0.98094345 |
| Body mass index | rs7200543 | 16 | 15129970 | *NTAN1 & PDXDC1* | 4.44E-06 | 7.5E-7 | 3.4723E-05 | 0.00489842 | 0.00700391 | 0.98806294 |
| Body mass index | rs1379578 | 16 | 20034418 | *GPRC5B-GPR139* | 1.20E-07 | 1.9E-8 | 3.1557E-07 | 0.00061107 | 0.00051583 | 0.99887278 |
| Coronary artery disease | rs2519093 | 9 | 136141870 | *ABO-SURF6* | 1.19E-11 | 2.2E-6 | 1.746E-08 | 0.01290281 | 1.3357E-06 | 0.98709583 |
| Coronary artery disease | rs10883806 | 10 | 104713076 | *BORCS7-ASMT* | 6.80E-08 | 1.4E-8 | 8.9643E-07 | 0.0008215 | 0.00108913 | 0.99808848 |
| Coronary artery disease | rs4766578 | 12 | 111904371 | *ATXN2* | 2.83E-10 | 3.3E-6 | 2.5215E-07 | 0.0160536 | 1.5454E-05 | 0.98393069 |
| Coronary artery disease | rs12906125 | 15 | 91427612 | *FURIN* | 8.64E-08 | 9.9E-9 | 8.926E-07 | 0.00067965 | 0.00131071 | 0.99800875 |
| Coronary artery disease | rs2870463 | 16 | 75250717 | *CTRB1* | 4.01E-06 | 7.5E-6 | 0.00062142 | 0.02430153 | 0.02431214 | 0.95076491 |
| Hemoglobin A1c | rs9295474 | 6 | 20652717 | *CDKAL1* | 1.13E-06 | 2E-19 | 1.1537E-11 | 3.4018E-10 | 0.0328007 | 0.9671993 |
| Hemoglobin A1c | rs10830963 | 11 | 92708710 | *FAT3-MTNR1B* | 2.99E-09 | 1E-7 | 1.6352E-06 | 0.00155919 | 0.00104601 | 0.99739316 |
| HDL cholesterol | rs2275767 | 1 | 39945297 | *MACF1* | 2.02E-7 | 3.8E-6 | 1.2389E-05 | 0.01103913 | 0.00110859 | 0.98783989 |
| HDL cholesterol | rs1515098 | 2 | 227073854 | *NYAP2-IRS1* | 1.223E-15 | 2.3E-6 | 2.672E-13 | 0.00827358 | 3.2028E-11 | 0.99172642 |
| HDL cholesterol | rs2062432 | 3 | 123078079 | *ADCY5* | 2.563E-6 | 5.8E-6 | 0.00016083 | 0.01393198 | 0.0112516 | 0.97465559 |
| HDL cholesterol | rs1482852 | 3 | 156798294 | *LINC02029* | 6.34E-8 | 2E-42 | 2.1205E-20 | 5.4744E-17 | 0.00038719 | 0.99961281 |
| HDL cholesterol | rs806794 | 6 | 26200677 | *HIST1H3E-HIST1H1D* | 7.761E-6 | 8.1E-7 | 0.00013986 | 0.00438354 | 0.03078034 | 0.96469625 |
| HDL cholesterol | rs1936806 | 6 | 127451665 | *RSPO3* | 2.368E-9 | 0.000024 | 6.0021E-07 | 0.03164209 | 1.8368E-05 | 0.96833895 |
| HDL cholesterol | rs12525163 | 6 | 152040291 | *ESR1* | 1.524E-7 | 6.4E-20 | 9.2645E-14 | 1.0696E-10 | 0.00086542 | 0.99913458 |
| HDL cholesterol | rs34346326 | 7 | 73016181 | *BAZ1B* | 3.27E-7 | 2.3E-6 | 1.4414E-05 | 0.00825917 | 0.00172775 | 0.98999867 |
| HDL cholesterol | rs4871598 | 8 | 126459990 | *TRIB1* | 8.561E-6 | 2.9E-6 | 0.00031905 | 0.00913858 | 0.03341553 | 0.95712684 |
| HDL cholesterol | rs7076938 | 10 | 115789375 | *BORCS7-ASMT* | 1.712E-7 | 4.7E-18 | 1.2573E-12 | 1.3046E-09 | 0.00096282 | 0.99903718 |
| HDL cholesterol | rs2071305 | 11 | 47370957 | *MYBPC3* | 1.188E-9 | 0.000044 | 4.4761E-07 | 0.04443704 | 9.6252E-06 | 0.95555289 |
| HDL cholesterol | rs6589581 | 11 | 116828859 | *SIK3* | 2.258E-9 | 1E-5 | 3.4956E-07 | 0.01925033 | 1.7809E-05 | 0.98073151 |
| HDL cholesterol | rs4766578 | 12 | 111904371 | *ATXN2* | 1.098E-10 | 3.3E-6 | 1.1542E-08 | 0.01018897 | 1.1213E-06 | 0.98980989 |
| HDL cholesterol | rs11057251 | 12 | 123791227 | *SBNO1* | 4.293E-9 | 8E-6 | 5.5483E-07 | 0.01694466 | 3.2187E-05 | 0.9830226 |
| HDL cholesterol | rs8064216 | 16 | 67293155 | *PLEKHG4* | 1.099E-6 | 0.000012 | 0.00011278 | 0.02124757 | 0.00516701 | 0.97347264 |
| HDL cholesterol | rs11667352 | 19 | 33918423 | *PEPD* | 5.088E-6 | 5.1E-6 | 0.00027748 | 0.01281194 | 0.02092159 | 0.96598899 |
| Insulin secretion | rs2206734 | 6 | 20694884 | *CDKAL1* | 6.390e-10 | 2.4E-17 | 6.6051E-12 | 5.0588E-09 | 0.00130396 | 0.99869603 |
| Insulin secretion | rs11187144 | 10 | 94469980 | *HHEX-ECOC6* | 2.750e-08 | 5.8E-13 | 2.0422E-08 | 1.5653E-06 | 0.01287859 | 0.98711983 |
| Insulin secretion | rs10830963 | 11 | 92708710 | *FAT3-MTNR1B* | 1.260e-14 | 1E-7 | 2.6182E-09 | 0.00151716 | 1.7231E-06 | 0.99848111 |
| Insulin sensitivity | rs1515098 | 2 | 227073854 | *NYAP2-IRS1* | 3.374e-09 | 2.3E-6 | 7.2456E-05 | 0.00887243 | 0.00802781 | 0.98302731 |
| LDL cholesterol | rs2030746 | 2 | 121309488 | *LINC01101, GLI2* | 8.605E-9 | 1.5E-7 | 1.1595E-07 | 0.00169231 | 6.8397E-05 | 0.99823918 |
| LDL cholesterol | rs9461684 | 6 | 31253444 | *HLA-C-WASF5P* | 3.709E-7 | 0.000026 | 7.1598E-05 | 0.03291188 | 0.00209914 | 0.96491738 |
| LDL cholesterol | rs6989280 | 8 | 126508746 | *TRIB1* | 1.874E-17 | 5E-8 | 6.7362E-16 | 0.0008934 | 7.5333E-13 | 0.9991066 |
| LDL cholesterol | rs532436 | 9 | 136149830 | *ABO-SURF6* | 4.02E-30 | 2.5E-6 | 1.4952E-26 | 0.00864788 | 1.7141E-24 | 0.99135212 |
| LDL cholesterol | rs4766578 | 12 | 111904371 | *SH2B3* | 9.112E-9 | 3.3E-6 | 7.3295E-07 | 0.01014905 | 7.148E-05 | 0.98977874 |
| LDL cholesterol | rs516246 | 19 | 49206172 | *ARRDC5 & UHRF1* | 1.33E-13 | 3E-7 | 6.593E-12 | 0.00253176 | 2.5975E-09 | 0.99746824 |
| LDL cholesterol | rs6016373 | 20 | 39154095 | *DHX35-MAFB* | 7.947E-19 | 7.8E-10 | 3.278E-18 | 7.9309E-05 | 4.1329E-14 | 0.99992069 |
| LDL cholesterol | rs753381 | 20 | 39797465 | *ZHX3* | 3.571E-25 | 1.5E-7 | 1.0302E-22 | 0.00169242 | 6.077E-20 | 0.99830758 |
| Myocardial infarction | rs2519093 | 9 | 136141870 | *ABO-SURF6* | 3.53E-17 | 2.2E-6 | 4.3411E-12 | 0.01257269 | 3.4094E-10 | 0.98742731 |
| Myocardial infarction | rs7475853 | 10 | 104673097 | *BORCS7-ASMT* | 3.63E-07 | 1.3E-8 | 5.2123E-06 | 0.00076451 | 0.00676649 | 0.99246379 |
| Myocardial infarction | rs4766578 | 12 | 111904371 | *SH2B3* | 6.39E-11 | 3.3E-6 | 1.959E-07 | 0.01564353 | 1.2327E-05 | 0.98434395 |
| Myocardial infarction | rs8039305 | 15 | 91422543 | *FURIN* | 8.53E-08 | 4.9E-8 | 3.7613E-06 | 0.00158479 | 0.00236396 | 0.99604748 |
| Type 2 diabetes | rs1515098 | 2 | 227073854 | *NYAP2-IRS1* | 2.6E-7 | 2.3E-6 | 4.1783E-05 | 0.00956279 | 0.00430858 | 0.98608685 |
| Type 2 diabetes | rs11708067 | 3 | 123065778 | *ADCY5* | 1.4E-8 | 2.8E-26 | 2.0683E-17 | 4.0943E-14 | 0.00050491 | 0.99949509 |
| Type 2 diabetes | rs3887925 | 3 | 186665645 | *ST6GAL1* | 4.1E-8 | 7.5E-6 | 2.0855E-05 | 0.01867161 | 0.00109486 | 0.98021268 |
| Type 2 diabetes | rs7766070 | 6 | 20686573 | *CDKAL1* | 5.3E-30 | 1.8E-28 | 1.7574E-34 | 2.2935E-15 | 7.6626E-20 | 1 |
| Type 2 diabetes | rs516946 | 8 | 41519248 | *ANK1* | 1.5E-7 | 2.8E-10 | 1.6352E-07 | 5.6178E-05 | 0.00290221 | 0.99704145 |
| Type 2 diabetes | rs2488075 | 10 | 94490174 | *HHEX-ECOC6* | 1.3E-14 | 1.7E-14 | 3.9031E-15 | 2.2002E-07 | 1.774E-08 | 0.99999976 |
| Type 2 diabetes | rs10830963 | 11 | 92708710 | *FAT3-MTNR1B* | 2E-7 | 1E-7 | 5.7933E-06 | 0.00160935 | 0.00358108 | 0.99480377 |
| Type 2 diabetes | rs1727313 | 12 | 123640853 | *MPHOSPH9* | 1.2E-6 | 0.000016 | 0.00038046 | 0.02814519 | 0.01295704 | 0.9585173 |
| Total cholesterol | rs2030746 | 2 | 121309488 | *LINC01101, GLI2* | 3.603E-8 | 1.5E-7 | 2.9841E-07 | 0.00169885 | 0.00017533 | 0.99812553 |
| Total cholesterol | Affx-920461 | 6 | 31268142 | *HLA-C-WASF5P* | 1.23E-6 | 3.4E-6 | 4.4948E-05 | 0.01031877 | 0.00429214 | 0.98534414 |
| Total cholesterol | rs6989280 | 8 | 126508746 | *TRIB1* | 1.855E-21 | 5E-8 | 1.295E-19 | 0.00089704 | 1.4423E-16 | 0.99910296 |
| Total cholesterol | rs532436 | 9 | 136149830 | *ABO-SURF6* | 6.14E-26 | 2.5E-6 | 1.0554E-22 | 0.00867983 | 1.2053E-20 | 0.99132017 |
| Total cholesterol | rs7112937 | 11 | 116880456 | *SIK3* | 1.94E-8 | 7.7E-7 | 4.3927E-07 | 0.00439127 | 9.9584E-05 | 0.99550871 |
| Total cholesterol | rs4766578 | 12 | 111904371 | *SH2B3* | 2.392E-14 | 3.3E-6 | 4.3067E-12 | 0.01018698 | 4.1846E-10 | 0.98981302 |
| Total cholesterol | rs516246 | 19 | 49206172 | *ARRDC5 & UHRF1* | 9.133E-17 | 3E-7 | 6.792E-15 | 0.00254165 | 2.6655E-12 | 0.99745835 |
| Total cholesterol | rs143384 | 20 | 34025756 | *GDF5* | 2.991E-6 | 6.4E-7 | 3.8186E-05 | 0.00390705 | 0.00964089 | 0.98641387 |
| Total cholesterol | rs6016373 | 20 | 39154095 | *DHX35-MAFB* | 1.002E-17 | 7.8E-10 | 2.8529E-17 | 7.9662E-05 | 3.5809E-13 | 0.99992034 |
| Total cholesterol | rs753381 | 20 | 39797465 | *ZHX3* | 1.35E-20 | 1.5E-7 | 1.4915E-18 | 0.00169914 | 8.763E-16 | 0.99830086 |
| Triglycerides | rs4660293 | 1 | 40028180 | *MACF1* | 2.873E-7 | 7.7E-6 | 3.0289E-05 | 0.01649578 | 0.00180252 | 0.98167141 |
| Triglycerides | rs1515098 | 2 | 227073854 | *NYAP2-IRS1* | 4.173E-12 | 2.3E-6 | 4.9084E-10 | 0.00824659 | 5.9029E-08 | 0.99175335 |
| Triglycerides | rs9461681 | 6 | 31251040 | *HLA-C-WASF5P* | 2.716E-8 | 1.9E-6 | 1.5191E-06 | 0.00738332 | 0.00020418 | 0.99241098 |
| Triglycerides | rs7766106 | 6 | 127455138 | *RSPO3* | 5.448E-7 | 0.000026 | 0.00010937 | 0.03289219 | 0.0032046 | 0.96379384 |
| Triglycerides | rs9388766 | 6 | 130354855 | *L3MBTL3* | 1.515E-6 | 9.2E-8 | 1.0855E-05 | 0.00126378 | 0.00850526 | 0.9902201 |
| Triglycerides | rs17401675 | 7 | 73057550 | *BAZ1B* | 8.469E-8 | 1.5E-11 | 4.6988E-09 | 7.9496E-06 | 0.00059072 | 0.99940133 |
| Triglycerides | rs6989280 | 8 | 126508746 | *TRIB1* | 4.173E-12 | 5E-8 | 5.3214E-11 | 0.00089405 | 5.9467E-08 | 0.99910589 |
| Triglycerides | rs7112937 | 11 | 116880456 | *SIK3* | 1.044E-7 | 7.7E-7 | 3.1399E-06 | 0.00437475 | 0.00071408 | 0.99490804 |
| Triglycerides | rs4985124 | 16 | 15125441 | *NTAN1 & PDXDC1* | 2.33E-7 | 4.2E-7 | 4.6494E-06 | 0.00307543 | 0.00150486 | 0.99541507 |
| Triglycerides | rs8182584 | 19 | 33909710 | *PEPD* | 1.365E-6 | 0.000032 | 0.00028692 | 0.03679828 | 0.00744996 | 0.95546484 |
| Triglycerides | rs132972 | 22 | 38562056 | *PLA2G6* | 2.85E-7 | 0.000031 | 6.6275E-05 | 0.03636444 | 0.00175294 | 0.96181634 |
| Waist circumference | rs6763927 | 3 | 141140366 | *ZBTB38* | 1.2e-06 | 0.000028 | 0.00020192 | 0.03702525 | 0.00522206 | 0.95755078 |
| Waist circumference | rs806794 | 6 | 26200677 | *HIST1H3E-HIST1H1D* | 1.5e-09 | 8.1E-7 | 1.7133E-07 | 0.00511774 | 3.3304E-05 | 0.99484878 |
| Waist circumference | rs1759645 | 6 | 34194866 | *GRM4-HMAG1* | 1e-05 | 6.7E-8 | 3.3193E-05 | 0.00120998 | 0.02666738 | 0.97208945 |
| Waist circumference | rs7766106 | 6 | 127455138 | *RSPO3* | 3.4e-06 | 0.000026 | 0.00042589 | 0.03531824 | 0.01148902 | 0.95276685 |
| Waist circumference | rs12704673 | 7 | 93154321 | *CALCR* | 9.3e-06 | 4.4E-6 | 0.00033657 | 0.0129663 | 0.02496405 | 0.96173308 |
| Waist circumference | rs7897654 | 10 | 104662458 | *BORCS7-ASMT* | 6.5e-06 | 6.1E-6 | 0.00030958 | 0.01566911 | 0.01906496 | 0.96495635 |
| Waist circumference | rs10784502 | 12 | 66343810 | *HMGA2* | 4e-07 | 3.8E-31 | 2.9621E-19 | 1.2544E-16 | 0.00235573 | 0.99764427 |
| Waist circumference | rs4985124 | 16 | 15125441 | *NTAN1 & PDXDC1* | 1.7e-07 | 4.2E-7 | 4.3338E-06 | 0.00352257 | 0.00122445 | 0.99524865 |
| Waist-to-hip ratio | rs17451107 | 3 | 156797609 | *LEKR1-CCNL1* | 1.2e-09 | 3.5E-42 | 8.35E-21 | 1.2408E-16 | 6.7292E-05 | 0.99993271 |
| Waist-to-hip ratio | rs13146972 | 4 | 145569692 | *GYPA-HHIP* | 6.5e-06 | 3.7E-11 | 5.6408E-07 | 1.6742E-05 | 0.03259328 | 0.96738941 |
| Waist-to-hip ratio | rs9491697 | 6 | 127456122 | *RSPO3* | 4.9e-10 | 0.000017 | 9.9375E-07 | 0.02821809 | 3.4222E-05 | 0.9717467 |
| Waist-to-hip ratio | rs11667352 | 19 | 33918423 | *PEPD* | 7.9e-07 | 5.1E-6 | 0.00010507 | 0.01431203 | 0.00718257 | 0.97840034 |
| Waist-to-hip ratio | rs143384 | 20 | 34025756 | *GDF5* | 3.5e-07 | 6.4E-7 | 1.8124E-05 | 0.00444749 | 0.00404041 | 0.99149398 |

**Table S6.** Overlaps between novel SNPs found to be associated with birthweight-coronary artery disease and birthweight-waist circumference and chromatin state, protein binding and other annotations from the Roadmap Epigenomics and ENCODE projects using the HaploReg tool (v4.1), and gene expression using GTEx

| **Cardiometabolic disease (CMD)** | **SNP (Gene)** | **Enhancer histone marks** | **DNAse** | **Motifs changed** | **eQTL hits**  (GTEx v. 6) | | |
| --- | --- | --- | --- | --- | --- | --- | --- |
|  |  |  |  |  | **Gene** | **Tissue** | **P-value** |
| Coronary artery disease | [rs2870463](http://archive.broadinstitute.org/mammals/haploreg/detail_v4.1.php?query=&id=rs2870463)  (*CTRB1-* *BCAR1*) | 5 tissues (Fetal adrenal gland, Placenta, Pancreas, HepG2 Hepatocellular Carcinoma Cell Line, H1 BMP4 Derived Trophoblast Cultured Cells) | Placenta, Pancreas | 5 altered motifs (MIF-1, Mxi1, Nanog, RFX5, SREBP) | *CFDP1* | Adipose - Subcutaneous | 2.90E-09 |
|  |  |  |  |  | *TMEM170A* | Skin - Sun Exposed (Lower leg) | 5.30E-09 |
|  |  |  |  |  | *CFDP1* | Adipose - Visceral (Omentum) | 4.80E-08 |
|  |  |  |  |  | *TMEM170A* | Skin - Not Sun Exposed (Suprapubic) | 1.40E-07 |
|  |  |  |  |  | *RP11-252K23.2* | Heart - Left Ventricle | 9.90E-07 |
|  |  |  |  |  | *CFDP1* | Skin - Sun Exposed (Lower leg) | 1.30E-06 |
|  |  |  |  |  | *CFDP1* | Whole Blood | 1.30E-05 |
|  |  |  |  |  | *CFDP1* | Cells - Transformed fibroblasts | 3.40E-05 |
|  |  |  |  |  | *CFDP1* | Adipose - Subcutaneous | 2.90E-09 |
| Waist circumference | rs12704673  (*CALCR*) | - | - | TCF4 | - | | |

**References**

1. Horikoshi M, Beaumont RN, Day FR, Warrington NM, Kooijman MN, Fernandez-Tajes J, et al. Genome-wide associations for birth weight and correlations with adult disease. Nature. 2016;538(7624):248-52.

2. Locke AE, Kahali B, Berndt SI, Justice AE, Pers TH, Day FR, et al. Genetic studies of body mass index yield new insights for obesity biology. Nature. 2015;518(7538):197-206.

3. Shungin D, Winkler TW, Croteau-Chonka DC, Ferreira T, Locke AE, Magi R, et al. New genetic loci link adipose and insulin biology to body fat distribution. Nature. 2015;518(7538):187-96.

4. Replication DIG, Meta-analysis C, Asian Genetic Epidemiology Network Type 2 Diabetes C, South Asian Type 2 Diabetes C, Mexican American Type 2 Diabetes C, Type 2 Diabetes Genetic Exploration by Nex-generation sequencing in muylti-Ethnic Samples C, et al. Genome-wide trans-ancestry meta-analysis provides insight into the genetic architecture of type 2 diabetes susceptibility. Nat Genet. 2014;46(3):234-44.

5. Dupuis J, Langenberg C, Prokopenko I, Saxena R, Soranzo N, Jackson AU, et al. New genetic loci implicated in fasting glucose homeostasis and their impact on type 2 diabetes risk. Nat Genet. 2010;42(2):105-16.

6. Soranzo N, Sanna S, Wheeler E, Gieger C, Radke D, Dupuis J, et al. Common variants at 10 genomic loci influence hemoglobin A(1)(C) levels via glycemic and nonglycemic pathways. Diabetes. 2010;59(12):3229-39.

7. Prokopenko I, Poon W, Magi R, Prasad BR, Salehi SA, Almgren P, et al. A central role for GRB10 in regulation of islet function in man. PLoS Genet. 2014;10(4):e1004235.

8. Walford GA, Gustafsson S, Rybin D, Stancakova A, Chen H, Liu CT, et al. Genome-Wide Association Study of the Modified Stumvoll Insulin Sensitivity Index Identifies BCL2 and FAM19A2 as Novel Insulin Sensitivity Loci. Diabetes. 2016;65(10):3200-11.

9. Nikpay M, Goel A, Won HH, Hall LM, Willenborg C, Kanoni S, et al. A comprehensive 1,000 Genomes-based genome-wide association meta-analysis of coronary artery disease. Nat Genet. 2015;47(10):1121-30.

10. Willer CJ, Schmidt EM, Sengupta S, Peloso GM, Gustafsson S, Kanoni S, et al. Discovery and refinement of loci associated with lipid levels. Nat Genet. 2013;45(11):1274-83.
